# Critical evaluation of the reliability of DNA methylation probes on the Illumina MethylationEPIC BeadChip microarrays for dementia research

**DOI:** 10.21203/rs.3.rs-3068938/v1

**Published:** 2023-06-26

**Authors:** Wei Zhang, Juan I. Young, Lissette Gomez, Michael A. Schmidt, David Lukacsovich, Achintya Varma, X. Steven Chen, Brian Kunkle, Eden R. Martin, Lily Wang

**Affiliations:** University of Miami, Miller School of Medicine; Dr. John T Macdonald Foundation, University of Miami, Miller School of Medicine; the University of Miami Miller School of Medicine; the University of Miami Miller School of Medicine; University of Miami, Miller School of Medicine; the University of Miami Miller School of Medicine; University of Miami, Miller School of Medicine; Dr. John T Macdonald Foundation, University of Miami, Miller School of Medicine; Dr. John T Macdonald Foundation, University of Miami, Miller School of Medicine; University of Miami, Miller School of Medicine

**Keywords:** DNA methylation, Alzheimer&rsquo;s disease, dementia, probe reliability, EPIC array

## Abstract

**Background:**

DNA methylation (DNAm) has been implicated in many diseases including dementia. Array-based technologies offer a cost-effective and comprehensive approach for measuring DNAm on a genome-wide scale. However, the accuracy of DNAm measurements obtained using Illumina arrays can vary across different probes. Previous research has focused primarily on assessing the reliability of DNAm in younger subjects, and have compared duplicate samples between the 450k-450k or 450k-EPIC platforms, with limited investigations on EPIC-EPIC comparisons.

**Methods:**

We conducted a comprehensive assessment of probe reliability on the Illumina EPIC arrays using 138 duplicated blood DNAm samples from subjects older than 65 years in the Alzheimer’s Disease Neuroimaging Initiative (ADNI) study. To assess the reliability of each probe, we computed intraclass correlations (ICCs) for each probe. Both the magnitude and patterns of reliability in the EPIC-EPIC comparison were assessed. Furthermore, we also investigated the impact of probe reliability on the analyses of epigenome-wide association studies (EWAS).

**Results:**

Our findings revealed the reliability of probes on the EPIC arrays is higher than those of previous studies involving duplicate measurements on 450k-EPIC or 450k-450k arrays. Consistent with earlier research, we observed increased reliability in probes with substantial between-subject variances or average methylation beta values ranging from 0.2 to 0.8. Lower reliability was observed in type I probes or probes located within the promoter and CpG island regions. In addition, we found some probes can yield high ICC values despite significant disagreement in duplicate measurements, primarily due to their relatively high between-subject variance. To account for such discrepancies explicitly, we introduced a novel statistical measure called the modified ICC, which penalizes the ICC based on the half-width of the 95% confidence limits of agreement. Importantly, we found probe reliability has significant implications in various downstream analyses of EWAS, such as meta-analysis, differentially methylated regions analysis, and integrative analyses within the cross-tissue or multi-omics contexts.

**Conclusion:**

We developed a valuable resource for dementia research, providing crucial reliability information for probes on the EPIC array. This resource can be utilized to identify and prioritize high-quality probes, thereby minimizing the potential for false discoveries and maximizing the potential of EWAS.

## INTRODUCTION

DNA methylation (DNAm) is a widely studied epigenetic mechanism characterized by the addition or removal of a methyl group at the 5th position of a cytosine [1]. Alterations of DNAm levels have been implicated in many diseases, including Alzheimer’s disease [2–6]. Methylated DNA is relatively stable and can be easily detected, thus is a viable source of biomarkers [7]. Although whole-genome bisulfite sequencing is still too costly for large-scale epidemiological studies, array-based technologies offer a cost-effective and comprehensive approach for measuring DNAm profiles on a genome-wide scale. The Illumina Infinium HumanMethylation450 BeadChip and its updated version, the Infinium MethylationEPIC BeadChip, provide probes that target over 485,000 CpG sites and 850,000 CpG sites per sample, respectively [8, 9].

In recent years, several studies have examined the reliability of DNAm levels measured using Illumina arrays and found that the reliability of methylation levels varies across different probes [10–15]. However, previous research has focused primarily on evaluating the reliability of DNAm levels in younger subjects (Supplementary Table 1), leaving a gap in our understanding of the reliability of CpG probes in older subjects who are involved in dementia research. Furthermore, most previous studies have compared duplicate samples between the 450k-450k or 450k-EPIC platforms, and there is a lack of studies for EPIC-EPIC comparisons.

To address these critical gaps and assess the reliability of blood DNAm levels measured using the EPIC arrays in older subjects, we conducted an analysis using 138 duplicated blood DNAm samples from the Alzheimer’s Disease Neuroimaging Initiative (ADNI) study [16]. We compared both the magnitudes and patterns of reliability observed in the EPIC-EPIC comparison with findings from previous studies. Our study aims to provide valuable insights into the reliability of blood DNAm levels measured by the newer EPIC arrays in dementia research, where such information has been lacking. To improve the measurement of probe reliability, we proposed a new statistic, the modified intra-class correlation coefficient (ICC). We then evaluated the impact of probe reliability on the analysis of epigenome-wide association studies (EWAS). Higher reliability of methylation levels increases the likelihood of reproducible findings, which are essential for the development of biomarkers or identifying actionable targets; therefore, our study provides a valuable resource for future methylation analysis in dementia research.

## METHODS

### Study dataset

We analyzed a subset of whole blood DNAm samples generated by the Alzheimer’s Disease Neuroimaging Initiative (ADNI) study [16], in which the same blood samples were measured twice (technical replications). To create a dataset of samples from independent subjects, for each subject we selected the initial visit data from the longitudinal ADNI study. Our study included a total of 138 samples measured on 69 independent subjects who were over 65 years old at the time of the initial visit. To avoid confounding batch effects (see also Discussion), only duplicates measured on different methylation plates were analyzed. The ADNI study datasets can be accessed at adni.loni.usc.edu.

### Pre-processing of DNA methylation data

The DNA methylation samples were measured using the Illumina HumanMethylation EPIC beadchip, which includes more than 850,000 CpGs. We pre-processed the samples using the SeSAMe 2 pipeline described in Welsh et al. (2023), which was found to perform the best and produced the largest percentage of reliable CpG probes in a recent comparison of various pre-processing and normalization pipelines [17]. Supplementary Table 2 shows the number of CpGs at each pre-processing step.

First, we removed CpGs that overlap with single nucleotide polymorphism (SNP), non-CpG probes, cross-reactive probes [18], and probes located on X or Y chromosomes. Samples and probes were further filtered using the iterative Greedy-cut algorithm (with a *p*-value threshold of 0.01) in RnBeads R package, which iteratively removes the probe or sample with the highest fraction of unreliable measurements one at a time [19]. Next, we removed additional probes that had missing values in more than 5% of samples or were masked by the pOOBAH (*P*-value with out-of-band array hybridization) algorithm in SeSAMe R package in more than 20% of samples. Finally, we performed noob (normal-exponential using out-of-band probes) background correction and a non-linear dye-bias correction [20]. These analyses were performed using the RnBeads and SeSAMe R packages.

### Estimation of probe reliability

To estimate the reliability of the CpG probes, we computed intraclass correlations (ICCs) for each probe based on methylation beta values, which were measured on duplicates of blood samples collected from the same subject and at the same visit. ICC is defined as $\frac{\sigma_{b}^{2}}{\sigma_{b}^{2}+\sigma_{w}^{2}}$, where $\sigma_{b}^{2}$ is the between-subject variance and $\sigma_{w}^{2}$ is the within-subject variance. As recommended by Koo and Li (2016) [21], the ICC values were computed using a two-way random effect, absolute agreement, and single-rating model, as implemented in the icc () function of the irr R package.

As discussed below in Results, probes with large between-subject variance could yield high ICC values even in the presence of substantial differences between duplicate measurements. To explicitly account for the amount of disagreement, we proposed to assess reliability using a modified ICC, which is defined as ICC – Half-width of 95% confidence Limits of Agreement (HoLA). HoLA is calculated as $1.96\sigma_{d}$, where $\sigma_{d}$ is the standard deviation of the differences between the two duplicate measures. Smaller values of HoLA indicate greater agreement between methylation levels in duplicate samples, while larger values of HoLA indicate more disagreement.

### Comparison of reliability of probes with different characteristics

To compare the reliability of probes with different characteristics (e.g., type I probes vs. type II probes), we performed mixed effects model analyses using the lmerTest R package [22]. For each comparison, we fitted a mixed effects model with probe reliability as the outcome and probe characteristic (e.g., type I vs. type II probes) as the fixed effect variable. To account for correlations in probes on the EPIC array, we additionally included random effects for chromosomes, genes, and co-methylated clusters. The co-methylated clusters of probes were computed using the coMethDMR R package [23], with methylation beta values as input. Because both fixed and random effects are included, these models fall into the general class of linear mixed effects models. By including random effects, the mixed effects model acknowledges that the observations (i.e., probes) within the same random effect (i.e., chromosome, gene, or co-methylated cluster) are more similar to each other than to observations from different groups or clusters. This allows for a more accurate estimation of the fixed effects while properly accounting for the correlated structure of the data. We also assessed the relationship between reliability with mean and standard deviation of methylation beta values. The mean of beta values were computed using all samples; the standard deviations of beta values were computed after randomly selecting one sample from the two duplicate samples.

### Evaluating the impact of probe reliability on mQTL analysis, DNAm-to-gene expression correlations, and surrogate variables

We searched mQTLs for the CpG probes using the GoDMC database [24], which was downloaded from http://mqtldb.godmc.org.uk/downloads. To select significant blood mQTLs in GoDMC, we used the same criteria as the original study [25], that is, considering a cis *P*-value smaller than 10^− 8^ and a trans *P*-value smaller than 10^− 14^ as significant. For the DNAm-to-mRNA association analysis, we examined probes located in promoter regions (within ± 2k bp of the transcription start site; TSS) and distal regions ( > ± 2k bp of TSS) separately. Specifically, for CpGs located in the promoter region, we computed the Spearman correlations between CpG methylation and expression levels of the target genes. On the other hand, for CpGs in distal regions, we computed the Spearman correlations between CpG methylation and expression levels of ten genes upstream and downstream, following the approach used in previous integrative DNAm-to-gene expression analyses for probes in distal regions [26, 27]. Subsequently, we selected the most significant *P*-value for each probe and considered *P*-values less than 1 × 10^− 5^ to be statistically significant, as in several previous analyses of DNA methylation measured in blood samples [5, 16, 28]. To evaluate the reliability of estimated cell-type proportions, we computed major immune cell type proportions in blood, including B lymphocytes, natural killer cells, CD4 + T lymphocytes, CD8 + T lymphocytes, monocytes, neutrophils, and eosinophils using the EpiDISH R package [29].

## RESULTS

### The probes on EPIC arrays have higher reliability than those in 450k arrays

The estimated ICCs for individual probes are available for Sugden et al. (2020) [15], Logue et al. (2017) [12], and Bose et al. (2014) [10]. Therefore, we first compared the distributions of the ICCs in our EPIC-EPIC comparison with results from these studies. Overall, we found a substantial correlation between the ICC values estimated in our EPIC-EPIC comparison and those estimated in previous studies. Specifically, the Spearman correlations between the ICC values estimated in our study and those from Sugden et al. (2020) [15], Logue et al. (2017) [12], and Bose et al. (2014) [10] were 0.703, 0.724, and 0.729, respectively. Consistent with previous studies [14, 15], we observed similar ICC estimates when using either methylation M-values or methylation beta values (data not shown).

**Figure 1:**
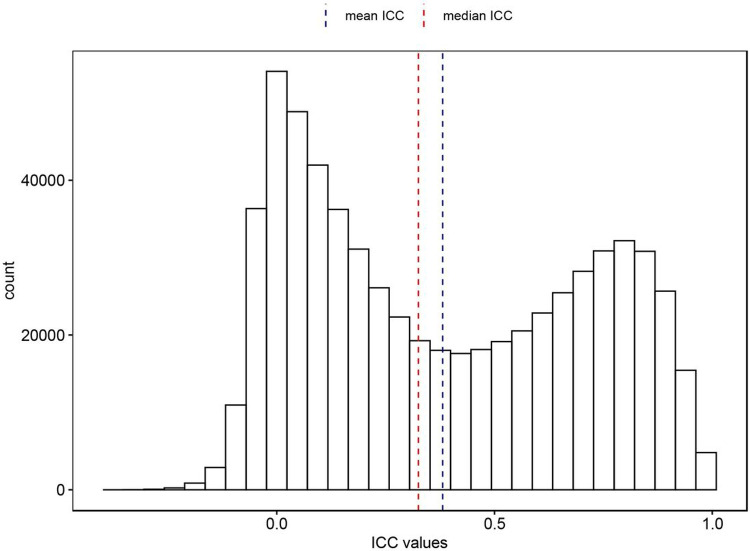
Distribution of estimated intraclass correlation coefficient (ICC) for 138 technical duplicate DNA methylation samples generated from 69 independent subjects with age older than 65 years in the ADNI study. Dashed line indicates median (red) and mean (blue) of ICC values at 0.325 and 0.381, respectively

The ICCs for probes in our EPIC-EPIC comparison ranged from − 0.362 to 0.999, with a mean of 0.381 and a median of 0.325 (Fig. 1). We found that these EPIC-EPIC comparisons resulted in higher ICCs than those of the 450k-EPIC comparisons in Sugden et al. (2020) [15], where the estimated ICCs for the 450k-EPIC comparison ranged from − 0.28 to 1.00, with a mean of 0.21 and a median of 0.09 (*P*-value < 0.001, Supplementary Fig. 1). The ICC values are generally interpreted as the following: < 0.4 (Poor), 0.4–0.6 (Fair), 0.6–0.75 (Good), and > 0.75 (Excellent)[30]. A comparison of the 333,588 common probes in both studies showed that a larger number of probes achieved good reliability (n = 38,528) or excellent reliability (n = 64,141) in our EPIC-EPIC comparison study, compared to the 450k-EPIC comparison in Sugden et al. (2020) study (good probes: n = 21,936, excellent probes: n = 18,865) (Supplementary Table 3). Overall, 214,951 probes (64.44%) had the same classification in both studies.

On the other hand, the ICCs in the EPIC-EPIC comparison are only slightly higher than those in the 450k-450k comparison estimated by Bose et al. (2014), which ranged from 0 to 0.998, with a mean of 0.366 and a median of 0.296 [10] (Supplementary Fig. 2). When comparing the 333,600 common probes in both studies, we found a larger number of probes with good or excellent reliability (good probes: n = 38,528, excellent probes: n = 64,144) in our EPIC-EPIC comparison compared to the 450k-450k comparison in Bose et al. (2014) study (good probes: n = 49,008, excellent probes: 40,001). Overall, 226,289 (67.83%) probes had the same classification in both studies (Supplementary Table 4).

### The distribution of ICC in the EPIC-EPIC comparison shows similar patterns to those obtained in the 450k-EPIC and 450k-450k comparisons

**Figure 2:**
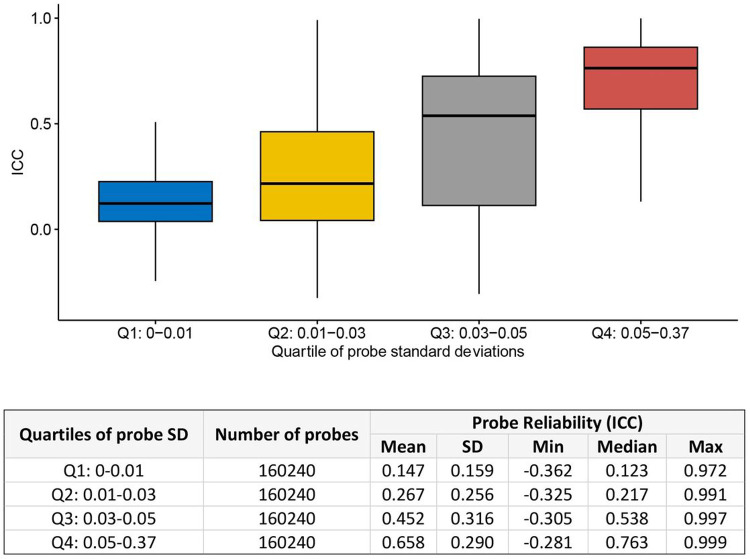
Probe reliability (ICC) increased as standard deviation (SD) of DNA methylation levels increased.

We found that the distribution of ICC values in our EPIC-EPIC comparison showed similar patterns to previous comparisons [10–13, 15]. Specifically, as indicated in the formula for defining ICC, we observed that ICC values increased with the between-subject variance $\sigma_{b}^{2}$ of the DNA methylation levels (Fig. 2). The highest ICCs values were observed for probes with average methylation beta values ranging between 0.2 and 0.8 (Supplementary Fig. 3). Additionally, ICC values were lower for probes located in CpG island and TSS200 regions (Supplementary Figs. 4–5). Type II probes on the EPIC array, which use a single sequence per CpG site to measure DNAm levels, showed significantly higher ICC values than type I probes, which use two separate sequences per CpG site (*P*-value < 2.2 × 10^−16^, Supplementary Fig. 6). Given that a substantial proportion of type I probes are located in CpG-rich regions such as CpG islands and promoter regions [9, 31], this result is consistent with the observed lower ICC values at CpG islands and promoter regions.

### The modified ICC explicitly accounts for agreement between duplicate DNAm measurements

**Figure 3:**
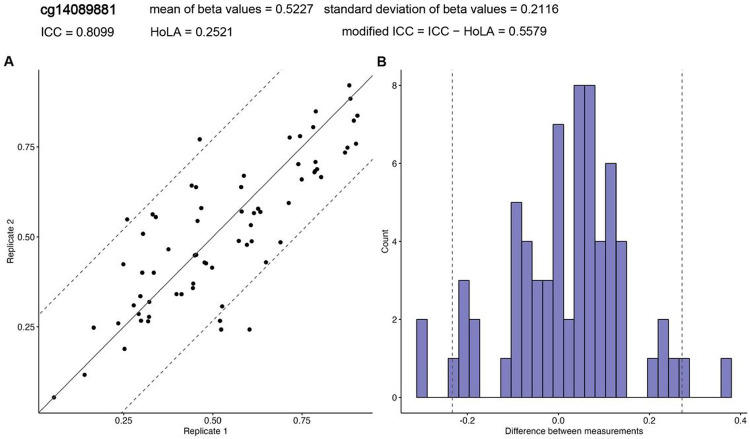
DNA methylation levels at cg14089881 for 69 pairs of duplicate samples in the ADNI dataset shows ICC value can be high (ICC = 0.8099) even when there is substantial disagreement in duplicate measurements (HoLA = 0.2521). In **(A)**, each point corresponds to methylation levels of duplicate samples from the same subject, and the centerline indicates perfect agreement. In both **(A)** and **(B)**, dashed lines indicate 95% confidence limits of agreement, which is computed as 1.96 x standard deviation of the differences between the two duplicate measurements. In **(B)**, shown is histogram of the differences in methylation levels in the duplicate samples. **Abbreviation:** HoLA = Half-width of Limits of Agreement

As noted previously [14, 32], a challenge when assessing the agreement of methylation levels in duplicate samples is that ICC is dependent on between-subject variance. Since ICC is defined by $\sigma_{b}^{2}/\left(\sigma_{b}^{2}+\sigma_{w}^{2}\right)$, its values can be strongly influenced by between-subject variances $\sigma_{b}^{2}$ of the methylation levels. Figure 3 shows the correlation plot for cg⁡14089881, which has an ICC value of 0.8099, indicating excellent reliability. For this probe, the estimated limits of agreement [33], or the 95% confidence interval for differences in methylation beta values in the duplicates, are (−0.233, 0.272), indicating considerable differences between the duplicate measurements. Note that the standard deviation (SD) for this CpG is 0.2116, which ranks 1166th (0.18%) largest among the 640,960 probes. Therefore, some probes, such as cg14089881, can achieve high ICC values due to their relatively high between-subject variance $\sigma_{b}^{2}$, even when there is substantial disagreement in duplicate measurements.

Therefore, we propose a modified ICC, that additionally considers the Half-width of the 95% confidence Limits of Agreement (HoLA). We define the modified ICC as ICC - HoLA, which penalizes ICC by the amount of disagreement in duplicate measurements for the probe. Note that the modified ICC prioritizes probes with a large ICC and a small HoLA. On the other hand, probes with a large ICC but also substantial disagreement in duplicate measurements will have a reduced modified ICC compared to the ICC. For example, the modified ICC for cg14089881 mentioned above is 0.5579, indicating only fair reliability for this probe after correction for disagreement based on HoLA.

Table 1 presents the probe classifications based on the modified ICC and demonstrates its good agreement with the original ICC, except for a small group of CpGs with substantial disagreement in duplicate measurements. The modified ICC retains the properties of the ICC, including similar patterns of association with mean and standard deviations of beta values, lower values in CpG islands and TSS200 regions, and at CpGs measured by type I probes (Supplementary Figs. 7–11).

In the following sections, we will assess the impact of probe reliability, as measured by the modified ICC, in relation to different types of analysis in epigenome-wide association studies (EWAS).

### Higher probe reliability is associated with the presence of mQTLs and significant correlations with downstream gene expression

We hypothesized that probe reliability might impact the effectiveness of integrative analyses that correlate DNAm with other types of omics variants, such as mQTLs or downstream gene expression. Methylation Quantitative Trait Locus (mQTLs) refers to genetic variations that influence patterns of DNAm. Min et al. (2021) performed a large mQTL study involving 32,851 subjects and found that approximately 45% of the DNAm sites on the Illumina array are influenced by genetic variants [24]. Supplementary Fig. 12 shows CpG probes with mQTLs have significantly higher reliability (*P*-value < 2.2 × 10^−16^). Specifically, CpG probes influenced by mQTLs have a median modified ICC of 0.546, compared to the other CpGs with a median modified ICC of 0.214.

Similarly, as DNAm is a key epigenetic modification that influences gene activity by regulating gene expression, we also investigated the impact of probe reliability on DNAm-to-mRNA correlations. In this analysis, we examined probes located in promoter regions (within ± 2k bp of the transcription start site; TSS) and distal regions ( > ± 2k bp of TSS) separately. For both groups of probes, we found the modified ICCs are higher for those probes significantly associated with downstream gene expression levels compared to other probes (*P*-value = 3.24×10^− 5^ and *P*-value = 1.41×10^− 9^, respectively; Supplementary Fig. 13). These findings highlight the importance of considering probe reliability in EWAS and their potential implications for understanding the relationship between DNAm, genetic variation, and gene expression.

### Higher probe reliability is associated with larger blood-brain DNAm correlations

For neurological disorders such as AD, it is preferable to use disease-relevant tissue for epigenetic studies. However, obtaining methylation levels in brain tissues from living human subjects is currently not feasible. As a practical alternative, measuring methylation levels in accessible tissues such as blood is often employed. Previous research by Hannon et al. (2015) examined matched DNAm profiles of pre-mortem blood samples and post-mortem brain tissues in the London dataset and found only a small proportion of CpGs showed significant brain-blood correlations in DNAm levels [34].

To assess the impact of probe reliability on cross-tissue associations, we examined modified ICCs for the probes in relation to the correlation of DNAm measured in the brain prefrontal cortex and blood in the London dataset. Supplementary Fig. 14 demonstrates that probes with higher brain-blood correlations in DNAm levels also had higher reliability. Specifically, for probes with high (> 0.75), medium (0.4–0.75), and low (< 0.4) brain-blood correlation, the median modified ICCs were 0.869, 0.786, and 0.215, respectively. These results are consistent with another recent study, which found an increase in reliability estimates in the 450k-EPIC comparison [15] for probes with moderate to high brain-blood correlations. These findings suggest that reliable probes, by providing more accurate representations of DNAm levels, could facilitate the identification of potential biomarkers associated with dementia.

### Higher probe reliability is associated with more consistent association signals in dementia studies

**Figure 4:**
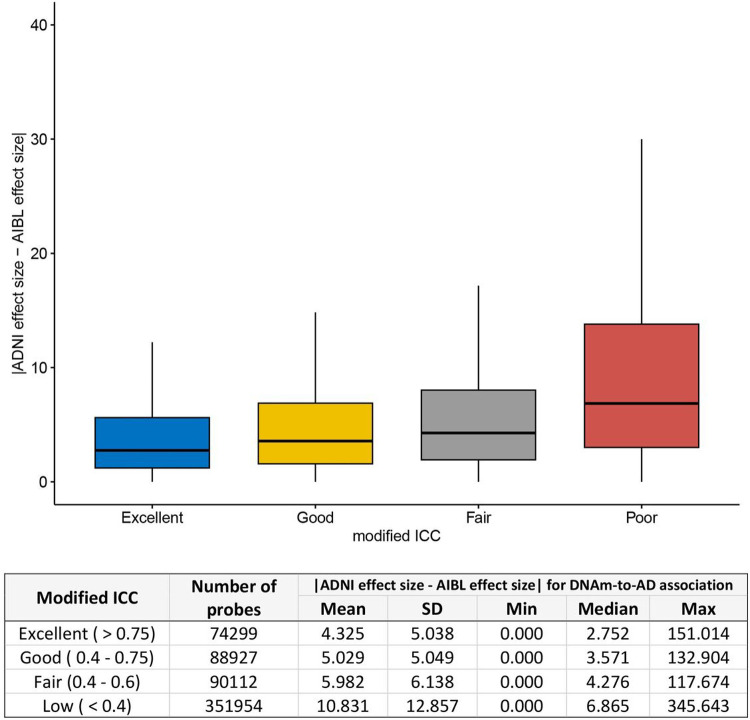
Higher probe reliability (modified ICC) is associated with smaller absolute difference in the estimated effect sizes of DNAm-to-AD diagnosis associations in ADNI and AIBL studies (*P* < 2.2 × 10^−16^). The effect sizes for DNAm-to-AD associations were obtained from Silva et al. (2022) (PMID: 35982059). Reliability of the probes were determined based on modified ICC: excellent (>0.75), Good (0.6–0.75), Fair (0.4–0.6), or Poor (< 0.4).

A previous study by Sugden et al. (2020) analyzed the effect of tobacco smoking on DNAm across 22 studies and observed that the number of replications of individual probes across studies positively correlated with reliability [15]. In the context of dementia research, we previously conducted a study on DNAm associated with AD diagnosis using two large clinical datasets generated by the ADNI and AIBL (Australian Imaging, Biomarkers, and Lifestyle) consortia. We hypothesized that poor probe reliability would impact the consistency of DNAm-to-AD associations estimated in the ADNI and AIBL datasets. Indeed, we found that the differences in estimated effect sizes for DNAm-to-AD associations between the two studies were smallest for probes with excellent reliability (modified ICC > 0.75), and the differences were largest for probes with poor reliability (modified ICC < 0.4) (Fig. 4). Furthermore, we also analyzed the results of our sex-specific study in AD [35] and found that the pattern of association between probe reliability and the consistency in estimated effect sizes was similar for both males and females (Supplementary Fig. 15).

**Figure 5:**
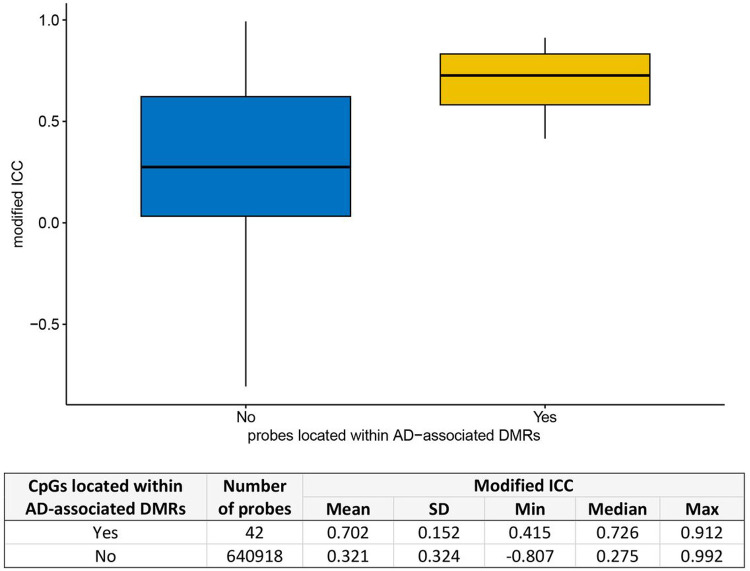
CpG probes located within differentially methylated regions (DMRs) had significantly higher probe reliability (modified ICC) compared to other probes (*P* = 0.049). The AD-associated DMRs were obtained from Silva et al. (2022) (PMID: 35982059).

Biologically, DNAm levels are often correlated across the genome, occurring as a regional phenomenon [36]. Differentially methylated regions (DMRs) refer to specific regions in the genome where the levels of DNAm consistently and significantly differ between different conditions. We also considered the impact of probe reliability on DMR identification. Interestingly, as shown in Fig. 5, we found that probes located within DMRs that were associated with AD exhibited significantly higher modified ICCs compared to other probes (*P*-value = 0.049). Specifically, the median modified ICC for probes within DMRs was 0.726, which was significantly higher than the median of 0.275 observed for other probes. We observed similar patterns of association in sex-specific analysis of AD-associated DMRs (Supplementary Fig. 16).

These findings highlight the importance of probe reliability in studying DNAm associations, as reliable probes help minimize discrepancies between different datasets and lead to more consistent results. Therefore, consideration of probe reliability is crucial for detecting genuine DNAm associations in dementia research.

### Surrogate variables for cell-type proportions are reliable

In EWAS, one common approach to account cell-type heterogeneity across different samples is to estimate the proportions of various cell types within each sample. These estimated cell-type proportions are then included as covariates in regression models. We assessed the reliability of these estimated cell-type proportions, and found they showed good agreement between the duplicate samples (Supplementary Table 5). Specifically, the modified ICC ranged from 0.693 to 0.930 across the different cell types. Notably, the proportions of NK (Natural Killer) cells and B cells were observed to have the lowest and highest reliabilities, respectively. These results are consistent with a previous study which examined DNAm levels in newborn samples and 14-year-old samples, which reported a high correlation in estimated cell-type proportions between duplicate samples [13]. Taken together, these findings provide strong support for using cell-type proportions to adjust for cell-type heterogeneity in EWAS.

## DISCUSSION

In this study, we performed a comprehensive evaluation of the reliability of probes on the Illumina EPIC arrays and created a valuable resource for dementia research (Supplementary Table 6). We carefully selected DNAm samples from the ADNI dataset, specifically focusing on independent samples from older subjects aged 65 years and above. To avoid batch effects and ensure an accurate assessment of probe reliability, we included duplicate samples that were placed on different methylation plates within the ADNI dataset.

Compared to previous studies that estimated probe reliability using duplicate measurements on either 450k-EPIC or 450k-450k arrays, the reliability estimates were higher in our study, in which both duplicates were measured using the EPIC arrays, consistent with results from previous smaller studies [15, 17] (Supplementary Table 1). Several factors could potentially account for the higher reliability estimates observed in our EPIC-EPIC comparisons. First, compared to the 450k arrays, the EPIC arrays include a significantly larger number of type II probes (~ 374,697) [9], which tend to have higher reliability than type I probes (Supplementary Fig. 6). Additionally, during the design of EPIC arrays, some probes from the 450k arrays that were found to be unreliable were removed [9, 37].

The source of DNA used in the reliability experiments may also play a role in our higher reliability estimate. Technical replicates for EPIC-EPIC and 450k-450k comparisons in most studies, including the ADNI study, were generated around the same time. However, replicates for 450k-EPIC comparisons were generated at a later time. We speculate that the 450k-450k and EPIC-EPIC comparisons may have utilized DNA extracted only once, while the DNA used in 450k-EPIC comparisons likely involved separate extractions. Therefore, reliability estimates in 450k-EPIC comparisons additionally account for variations in DNA extractions, potentially contributing to the lower reliability estimates observed in these comparisons.

It is important to consider additional factors that contribute to probe reliability. During our pre-processing step, we incorporated the pOOBAH (*P*-value with Out-Of-Band Array Hybridization) from the SeSAMe R package. This step specifically identifies and removes probes with hybridization issues [20]. Therefore, low reliability is unlikely to be attributed to failed probe hybridization to the target DNA as previously demonstrated [15]. However, an important factor that might impact reliability estimates are batch effects [10]. Notably, when we calculated reliability using duplicated samples placed on the same methylation plate, we observed an increase in the median ICC for the EPIC-EPIC comparison from 0.325 to 0.733. This highlights the impact of the batch effect on reliability estimates and emphasizes the importance of accounting for the batch effect in methylation studies.

Consistent with previous studies, we observed that reliability of the probes was influenced by the mean and variance of the methylation levels as measured by beta values [14, 32, 38]. To understand this, note that the ICC is defined by $\sigma_{b}^{2}/\left(\sigma_{b}^{2}+\sigma_{w}^{2}\right)$[14] where $\sigma_{b}^{2}$ and $\sigma_{w}^{2}$ are between-subject and within-subject variances, respectively. Therefore, the ICC is influenced by between-subject variances of the probes, and probes with low variation in methylation levels, corresponding to low between-subject variance, would result in low ICC values.

It is well-known that the mean and variance of methylation beta values follow an inverse-U relationship. If we consider the beta value as the proportion (p) of methylated cells in a large population of cells, the mean-variance relationship of beta values is consistent with the theory that for a binomial proportion p, the variance p(1-p)/n, where n is the total number of cells, is highest when p is 0.5. Therefore, between-subject variance peaks around a mean beta value of 0.5 and is lower for probes with the lowest and highest beta values [15].

Consequently, probes with extremely low or high average beta values have lower variances and are more likely to be classified as unreliable compared to probes with intermediate beta values. Consistent with previous observations in Xu and Taylor (2013) [14], we also found that among probes with very low or high average methylation beta values (below 0.1 or above 0.9), only a small portion (14.12% or 52,471 out of 371,531 probes) were classified as having good or excellent reliability (i.e., ICC > 0.6). In contrast, for probes with intermediate average methylation beta values, a significantly higher proportion (58.5% or 157,617 out of 269,429 probes) were classified as having good or excellent reliability. These results are consistent with our observation of lower ICC values in the TSS and CpG island regions, which are evolutionarily conserved in gene regulation [39].

On the other hand, probes with large between-subject variance could yield high ICC values even in the presence of sizable differences between duplicate measurements. To explicitly account for the disagreement in duplicate measurements, we proposed the modified ICC, which incorporates a penalty based on the half-width of the 95% confidence limits of agreement. We found that the modified ICC maintains the characteristics of the original ICC, with similar patterns of association with the mean and standard deviations of beta values. It also has lower values in CpG islands and TSS200 regions, as well as for CpGs measured by type I probes. The classification of probes based on the modified ICC largely agrees with the classifications based on the original ICC, except for probes with substantial disagreement in duplicate measurements.

We next studied the implications of probe reliability in EWAS, and our findings highlight its significant impact on downstream analyses. Our analyses revealed that probe reliability plays a crucial role in the success of integrative analyses involving DNAm and other types of omics data, such as mQTLs and mRNA gene expression. Furthermore, we observed that blood DNAm measurements obtained from probes with higher reliability are more likely to show concordance across different tissues in their associations with brain DNAm. Finally, we demonstrated that higher reliability is also associated with more consistent effect sizes and the identification of DMRs in dementia studies. This is likely because methylation signals from unreliable probes can be contaminated with noise, thus increasing the likelihood of generating false positives. Encouragingly, we found surrogate variables for cell-type proportions demonstrated good to excellent reliability across all major immune cell types in the blood, supporting the use of estimated cell-type proportions in addressing cell-type heterogeneity in EWAS.

Several limitations and areas of future study are in order. First, our analyses focused on methylation levels measured in whole blood, so the results may be applicable only to EWAS conducted using blood samples. Additional studies that assess the reliability of EPIC array probes in dementia research, such as those involving brain tissues and other accessible tissues (e.g., saliva), are needed. Second, due to the specific criteria we applied, including selecting independent subjects, restricting the age range to older than 65 years, and ensuring samples were placed on different methylation plates, we were able to include only a relatively small number of 69 pairs of duplicate samples in this study. Future studies with larger sample sizes are needed to replicate our findings. Finally, it is important to note that reliability estimates can be influenced by the choice of normalization procedures. In this study, we used the SeSAMe 2 pipeline, which was found to have the best performance and yielded the largest percentage of reliable CpG probes in a recent comparison of various pre-processing and normalization pipelines in a small study with 16 pairs of duplicate samples [17]. Additional studies with larger sample sizes are needed to thoroughly evaluate and compare different pre-processing procedures for estimating the reliability of DNAm levels in EPIC arrays.

To reduce the burden of multiple comparisons in EWAS, some authors have proposed excluding probes with low reliability *a priori* [10, 40], while others cautioned against this approach, as it may potentially exclude probes with low variability that are located in important gene regulatory regions [14]. An interesting topic for further research is designing step-wise multiple comparison procedures that maximally leverage reliability information of all the probes, while controlling overall Type I error rate in EWAS. The idea is to test all the probes, but with the most reliable probes first, and appropriately adjust the significance level of each probe analysis, to account for the increased chance of obtaining a false positive result when conducting multiple comparisons.

## CONCLUSION

We comprehensively evaluated the reliability of probes on the EPIC arrays. We observed that probes with substantial between-subject variance could still yield high ICC values, even when there were notable differences between duplicate measurements. To explicitly account for disagreement between duplicate measurements, we proposed a new statistical measure, the modified ICC, which penalizes the ICC by half-width of the 95% confidence limits of the agreement. Our findings revealed that probe reliability has significant implications for various downstream analyses of EWAS. Importantly, we have generated a valuable resource for dementia research by identifying a set of high-quality probes on the EPIC array, which will contribute to optimizing the robustness and potential of EWAS.

## Figures and Tables

**Table 1:** Comparison of probes reliability classifications based on ICC and modified ICC, which is computed as ICC - HoLA, where HoLA refers to Half-width of Limits of Agreement. The probe classifications based on modified ICC agree well with the classifications based on the original ICC, except for a small group of probes (highlighted in red) with substantial disagreement as measured by HoLA in duplicate measurements.

Class based on ICC	Class based on modified ICC			
	Poor	Fair	Good	Excellent
**Poor**	349,650 (100%)	0 (0%)	0 (0%)	0 (0%)
**Fair**	24,287 (29.9%)	56,935 (70.1%)	0 (0%)	0 (0%)
**Good**	15 (0%)	37,011 (43.6%)	47,895 (56.4%)	0 (0%)
**Excellent**	0 (0%)	200 (0.2%)	44,800 (35.8%)	80,167 (64%)

## Data Availability

The ADNI datasets can be accessed from http://adni.loni.usc.edu. The scripts for the analysis performed in this study can be accessed at https://github.com/TransBioInfoLab/DNAm-reliability
